# Thirty Years' Progress in the Study of Rheumatic Heart Disease
*An advanced Lecture in Medicine read in the Medical School of University College Hospital on 14th March, 1933, by Dr. C. Bruce Perry.


**Published:** 1933

**Authors:** Carey F. Coombs


### THIRTY YEARS' PROGRESS IN THE STUDY OF RHEUMATIC HEART DISEASE.*

BY THE LATE

CAREY F. COOMBS, M.D., F.R.C.P.

This lecture is printed as Dr. Carey Coombs had prepared it. It will be noticed that some of the paragraphs are personal remarks addressed to the Chairman, Dr. F. J. Poynton.

---

THIRTY years—to the very month—have passed since my election to the Medical Registrarship at St. Mary's Hospital. It is difficult to realize this, for I remember as clearly as if it had been yesterday that within the first few days of that invaluable experience I was waylaid by you, sir, at the door of De Hirsch Ward and—to my great delight—invited to interest myself in the work that you were doing then. I had already served as a clerk to Dr. Cheadle and House Physician to Dr. Lees; and had, moreover, been deeply interested by the work then being carried on by Dr. Paine and yourself; so that your invitation fell on ground that was ready to receive it. Whether the fruit that it has borne will be of value to anyone else is debatable; but I have no doubt that it has been of the greatest value to me. The subject furnished me with an introduction to cardiology, to the study of disease in children, to bacteriology and

* An advanced Lecture in Medicine read in the Medical School of University College Hospital on 14th March, 1933, by Dr. C. Bruce Perry.

morbid histology; so that the grain of mustard seed has given me a tree with many branches in which ideas of all kinds have found a lodging.

If I may for a moment continue to be reminiscent, the first step that I took, on your advice, sir, was that of analysing clinical notes, chiefly from Sir William Broadbent's old case books. Of the lessons taught by this study one in particular will serve as an opening to this address; namely, the changing character of the clinical picture of this disease. In those notes, drawn from the closing decades of last century, the type of case that predominated was the acute rheumatism of the young adult that was already, early in this century, becoming less common. This tendency has been maintained. We rarely see nowadays the adult type of acute rheumatism, with high fever, painful swelling of many joints, and severe but evanescent carditis. Rheumatic hyperpyrexia is almost unknown to us to-day, and pericardial friction is much less often heard than it was.

The main interest has steadily shifted to the rheumatic child with cardiac lesions. This is altogether to the good, for it is the cardiac lesions that matter, and it is in childhood that those lesions are most prevalent and most dangerous. One result of this changing outlook is, however, less fortunate, and that is the lack of a satisfactory nomenclature. We have learnt that this is an infective disease, and that its chief feature is a cardiac inflammation which may be complicated by various symptoms, especially in childhood, and by polyarthritis especially in adults. As Norman Moore put it in his Lumleian Lectures, it is a pity that we did not know this from the first, as we might then have labelled the disease cardiac and not rheumatic fever. In France the same difficulty is experienced, and while there is a natural tendency to take refuge in the term "maladie de Bouillaud," after the versatile physician who described the disease so thoroughly a hundred years ago, we shall, I think, have more sympathy with the proposal of Bezançon and Weil, to reserve the term "maladie rhumatismale" for that which Dr. Reginald Miller has in this country, from the pediatric angle, designated "juvenile rheumatism." After all, this has the merit of following the course of events which has been all in the direction of separating different ætiological groups from the amorphous mass of disease vaguely covered by the term "rheumatism," and leaving that word to be used only for the group of which we are to think to-day. At all events, I have found it convenient to speak of cardiac rheumatism, just as one has grown accustomed to speak of pulmonary tuberculosis and cerebral syphilis.

This digression upon a matter of nomenclature is only justified by one fact; namely, the international nature of this disease. Already we have had a French view of the matter, and to this I add Alvin Coburn's "Rheumatic State" from New York. The one common feature is the word "rheumatism." Let us call the whole thing "rheumatism," qualifying this by adjectives such as juvenile, cardiac and so forth, where these are necessary.

But if this term is to be limited to a particular disease, what is that disease? Its individual mark is its capacity to produce a certain definite cardiac lesion, a pancarditis. Though this fact has been taught as fundamental by Dr. Poynton and others for over thirty years, it is still in danger of being mutilated by the use of such limited terms as rheumatic pericarditis, rheumatic endocarditis. These terms tell the truth, but not the whole truth. The characteristic feature of the cardiac lesion is that all parts of the heart—valves, muscle, and serous investments — are simultaneously damaged. This fact has been fully elaborated by many workers since that paper published in 1899 in which you, sir, first described inflammatory foci in the myocardium. A few years later came the recognition, by Professor Aschoff, of the peculiar and characteristic nature of these foci, and this led us to perceive what I think had been hidden from us until then, that the basis of all these changes was a vascular basis. Further work has confirmed this, and proved that not in its acute phases only, but also in the chronic period, the whole range of the cardiac lesions is based on the coronary supply. Whether we tap that supply at the head or in the twigs one finds the same thing, an inflammatory reaction, mainly productive and tending to chronicity, attacking not only the connective tissue in the vicinity of the smallest twigs, but also the very trunks themselves (Perry). And what is true of the cardiac wall is true also of the aorta at its root, of the synovial membranes of the joints, of the pia mater, of the kidneys (Pappenheimer and Glahn), and also of that most rheumatic of all lesions, the subcutaneous node. The more one studies the histology of this the more does it appear to be vascular in its origin; to arise, in fact, from the impact of the infective agent upon an arteriole within whose lumen it has been detained. The most recent addition to this category of blood-borne lesions is the "rheumatic lung." Both Naish of Sheffield and Fraser of Bristol have figured inflammatory foci seen in the lungs of children dying of acute cardiac rheumatism which appear to conform in essentials to the Aschoff nodule. Fraser, in common with Shaw and others, has also described similar foci in the neighbourhood of the rheumatic tonsil. There is, then, a steady accumulation of evidence to support a belief in arteriolitis as the essential lesion of rheumatism. And it is in the heart that this lesion finds its most characteristic as well as its most formidable expression.

This statement I make by way of justifying the basis which we adopted for certain recent researches into the ætiology of this disease. About fifty years ago a Bristol physician, Dr. Budd, the grandfather of my friend and colleague Mr. John Wright, making researches into the causation of typhoid fever, was constrained to use spot-maps ; the first time, I believe, that this device had been used. That we used the same device was, I am afraid, no more than a coincidence ; except that I have always felt that cities of moderate size, such as Bristol with its 400,000 odd inhabitants, furnished an excellent field for such inquiries. But this fact was not the only one that commended our area to those who were interested in such researches. We also had the ideal periphery in that it included seaboard, hillside and fenland ; industrial as well as rural communities ; limestone as well as chalk ; and so on. We were also fortunate in being able to organize a team of medical men who took up the work with enthusiasm. Let me say here, in parenthesis, practical experience of the possibilities of voluntary co-operation between clinicians on the one hand and the State medical services on the other has convinced me that it is in this direction that we must look for a solution of our problem of organization, to a judicious blend, that is to say, of established order with individual enterprise.

Now, one of our difficulties was to define our unit. In other words, when we marked a dot on the map what was to be the exact meaning of the dot ? How was one to define the disease under inquiry so as to reduce the margin of error to a minimum ? One thing on which we agreed without much difficulty was to limit our inquiry to individuals with cardiac lesions. Our other limitations I do not propose to discuss ; but on this one it is worth while to dwell, since it proves that when you subject this area of disease, with its rather nebulous margins, to the test of asking what is unmistakably and indisputably rheumatic, it is the cardiac lesion that you select.

Now, there are many points of ætiological interest that emerge from any study of the subject that accept this basis. Some of these are not in dispute. For example, I do not suppose that, in this country at all events, its predilection for the decade 5–15, and for girls rather than for boys, would be disputed. I have wondered whether the age incidence is the same in America, but in the other countries of Northern Europe both these generalizations appear to hold good.

Before passing on to some of the other factors unearthed or confirmed by this inquiry, I am going to allude to a negative fact which may, I think, prove to be important, and that is the freedom of all other animals from this disease. For some years past we have been trying to get hold of specimens of cardiac infection from other animals. Thanks to the kindness of veterinary colleagues, and also of pathologists attached to the Zoological Gardens of London and Bristol, we have been able to survey this field, I will not say exhaustively, but at all events broadly. The lesions which we have encountered have been and will be elsewhere described. Here it is enough to say that we have nowhere met anything resembling cardiac rheumatism. Information given to me by Sir Thomas Lewis with regard to certain mitral scleroses in Dutch dogs led me to go to Holland and to interview Professor Schornagel of Utrecht, whose experience of disease in dogs is unrivalled. He showed me specimens of the lesion in question, but (although I have not yet had a specimen to examine microscopically) the naked-eye appearances are not those of cardiac rheumatism. They suggest a degenerative rather than an inflammatory process, and form no exception to the rule that cardiac rheumatism occurs only in man.

Now, before returning to our Bristol investigations let me speak briefly of other data which were revealed by this same visit to Holland. First, I was able to satisfy myself by conversations with Professor Christina de Lange and Dr. Stam at the Binnen-Gasthuis in Amsterdam that the juvenile rheumatism which they see there is exactly the same as that which we see here. In fact, on the morning when I called on Professor de Lange she had just been lecturing on subcutaneous nodes, and showed me some beautiful pictures. A second point which Dr. Stam's figures of admissions to hospital proved to me was that this disease constitutes a much smaller fraction of the total number of in-patients admitted to their children's wards than would be the case in London; and this in spite of the fact that Amsterdam is built on an archipelago—islands scattered in a maze of canals—the wettest city in Europe, not excepting Venice. But this question of the world distribution of rheumatic heart disease deserves a more careful scrutiny than it has as yet received. I do not think certain broad generalizations, for example, that in Europe the disease prefers the countries that border on the North Sea, are likely to be disturbed. However, there is much to be gained, and nothing to be lost, by an international study of this matter, and you will be glad to know that preliminary steps in this direction have already been taken.

Now this reminds me of an analogy which may perhaps serve at this point to bring us back to our Bristol studies. Much of the time which I spent in years gone by on this disease was devoted to its morbid histology, and I remember clearly the look of the heart in which I first encountered the foci that Aschoff (unknown to me at the time) had already described. It was that of a young adult, and showed not only the usual and obvious fibrinous deposits both on valves and pericardium, but also a very definite dilatation of the ventricles. This superficial survey, I suggest to you, is the analogue of that glance that we have just taken at the map of Europe.

But if we are to know more we must look closer. In the case of the heart one had to cut out blocks, make and stain sections, and examine these under the microscope. The case of the map is not different, except that whereas it is possible to take many blocks from the heart for histological examination, it has so far been possible to take a few areas only for closer inspection. London (Kensington) was treated in this way by Fenton and Lightwood in 1927–28; Birmingham by Thomson in 1925–26; New York by Coburn in 1929; and Bristol by ourselves in 1927–31. Now, whether we think of our problem in terms of geography or of histology, it is the same kind of fact that we find; namely, a concentration within particular areas of that departure from the normal which we call a "disease." Further reflection serves to carry the analogy a little deeper, for in the map, as in the section, it is not the cause of the disease that we see, but the reaction to that disease. Please remember that I have so far not gone beyond a low-power examination of these areas. At this stage it is the reaction that we are studying, but we are trying to learn from that reaction something about the nature of the disease. And what is there that we can claim to have learnt ? If I speak first of the Bristol facts, please do not think me indifferent to the value of those unearthed elsewhere ; it is only that those with which one is most familiar naturally impress one most. From a low-power study of that area two facts stand out. The first, a positive one, is that in the City of Bristol the incidence of this disease was five times as great as in the rural areas which surround it. The second, a negative fact, is of no less significance ; namely, the absolute unimportance of physiographic differences—geology, altitude, contour and so on. All these were powerless to produce any particular localization of the disease within a period of three years. Whether this contrast between city and country holds good for other areas remains to be seen. Thanks to the help given by the Harmsworth Trustees, it has been possible to enlist the help of workers in other cities, and I have no doubt that when their data are assembled we shall derive from them results of the highest value. At the moment, however, I do not think we ought to go beyond this, that so far as our own area is concerned the disease is a disease of the big city.

Now, when we look under a rather higher power at this big city, this focus of infection, what do we see ? The answer is decisive up to a point ; namely, an almost complete absence of the disease from certain areas and its concentration in others. These contrasts are susceptible of two kinds of explanation. That which has been generally adopted, in one form or another, regards them as expressions of a different soil. The reasons for this preference are twofold. In the first place, most of the thought devoted to the subject of rheumatic infection, in recent decades at all events, has emanated from pædiatrists, who are naturally apt to be preoccupied with the soil rather than with the seed. Then, again, we have been taught by the bacteriologists to indict for this offence a group of micro-organisms which is so diverse in the total range of its misdeeds that one cannot but think circumstances largely responsible for this versatility. These influences have kept our attention focused on the soil rather than the seed.

Now, however, we are being asked to consider more respectfully an alternative view; namely, that which interprets the concentration of cases in a particular area as a direct expression of saturation with micro-organisms. Here again we can discern two influences at work. The first is that of preventive medicine. Our public health colleagues have interested themselves in the question of rheumatism since the days of Newsholme, but within recent years, thanks largely to the influence of Alison Glover and the impetus given to this movement by Reginald Miller's address to the British Medical Association at Portsmouth in 1923, this interest has been greatly intensified, and I myself owe more than I can tell to the stimulation and help which I have received from my public health colleagues in the West of England.

But this is not the only breath of fresh air that has awakened us to a realization of the opening that lies before preventive medicine in this field. There is a breeze blowing across the Atlantic from the shores of New England that has helped to stir up minds and thoughts that were perhaps in danger of stagnation. I am thinking particularly of the younger generation of Americans, Alvin Coburn in New York and J. R. Paul in Connecticut. These, with our own epidemiologists, are telling us that we must at least consider concentration of the infective agent as a factor determining the incidence of the disease.

This brings us back to the crux of the dilemma —is it the seed or the soil that matters ? The answer is that it is both. We are too apt to think of what we call diseases as clearly-defined phenomena, with sharp, clear-cut edges. That this is not true of rheumatism is only too obvious. Even the cardiac lesions, which we have chosen as lying at the centre of the rheumatic area, present difficulties ; for example, that of deciding where rheumatic carditis leaves off and ulcerative endocarditis begins. And if we pass to other parts of the area, for example to the articular province, we shall find the difficulties confronting our boundary commission greatly increased. Who is to say when a polyarthritis ceases to be rheumatic and becomes rheumatoid ? As Coates has shown, there is some association between cardiac disease of the rheumatic kind and rheumatoid arthritis. The same thing is true of that most "rheumatic" of all lesions, the subcutaneous node. Coates and I compared specimens taken from rheumatoid patients, from a man with chronic ulcerative endocarditis, and from straightforward cases of rheumatic carditis ; and we found them histologically indistinguishable from one another. It is clear from these considerations that the edge of the rheumatic territory is not a hard black line, but a blur of many colours.

From this we may deduce a multiple ætiology. This bunch of phenomena that we call a disease is the outcome of interplay between many factors. It is true that these are still divisible into seed and soil, hammer and anvil, subject and object; but we are not to be surprised if we discover variabilities in each of these two groups.

But before glancing at these variations let us find out what is established and relatively constant. Now, to borrow another analogy from the histological picture, it is sometimes easier to discern what is happening at the edge of a focus than at its middle. Translating this into terms of our West of England area, I have found it difficult to learn much as yet from our map, except for the striking distinction between town and country of which I have already spoken, and a remarkable immunity of the well-to-do suburbs from the disease.

Now, it happened that during this field research there were two outbreaks of rheumatism in boys' schools. I saw something of both, and can bear witness to their essential similarity. One of them was thoroughly studied and carefully described by W. H. Bradley. Both outbreaks attacked boys living in good circumstances—well fed and housed, and in the country, well away from city conditions. In both instances waves of infection of the upper air passages by hæmolytic streptococci were followed, at an interval of about a fortnight, by the development of acute rheumatism in a percentage of the infected boys, with evidence of carditis in most cases. There was no doubt as to the widespread prevalence of hæmolytic streptococci; but only a proportion of those from whom these organisms were grown showed local signs—tonsillitis, etc.—and of those who showed these local signs only a small percentage developed rheumatism. That is to my mind the most significant fact that emerges from these observations; and as it is corroborated by other experiences (Schlesinger, Sheldon and Collis), it may, I think, be accepted as a fact that of those who are infected by these streptococci only a few develop rheumatism.

Now, before applying this fact, exposed to us by the examination of the edge of the focus, let us assimilate another fact which these same experiences have brought to light; namely, that of the incubation period of rheumatism. An interval of about a fortnight elapsed between the throat infection and the onset of rheumatism, and this agrees well enough with experiences gathered from cardiac convalescent homes by Schlesinger, and Collis and Sheldon in this country and by Coburn in the United States. We shall need to notice this fact again presently. Now, however, let us see whether we can apply the other great lesson of these epidemics to the rheumatism which is endemic in our great cities.

As we have seen, this is particularly concentrated in certain areas. There is little doubt that this is determined to some extent by environment. A survey of the streptococcal content of a great city would be a colossal task, and I do not think anyone has attempted it. The nearest approach of which I know is that of Coburn who studied the throat flora of three groups of patients in New York for twelve months. All these cases showed a basic flora of streptococcus viridans, but there was also a considerable incidence of hæmolytic streptococci. This varied from month to month, but the variations in each group were roughly the same. The moral of these observations is that between one area and another within the same city the streptococcal content does not vary anything like so widely as the incidence of rheumatism does. For example, the ward of Bristol which heads the list has twenty times as many cases per head of population as that at the bottom. Some of this difference is surely due to contrasts in environment. Probably these affect not only the health of the host but also the concentration of the infection. The pall of smoke that hangs over our great cities is significant not only because it cuts off the sun's rays, but also because it indicates defective ventilation by currents of wind; and I suppose there is no more powerful diluent of bacteria than these are. Glover in his Milroy Lectures brought forward much evidence supporting a belief in droplet infection, and stressed its dependence on defective ventilation.

However, we cannot leave this airy hypothesis to explain away all that is to be ascribed to environment. Much time and thought have been spent in a search for other factors. Housing especially has been accused, and in the attempt to find something which preventive medicine can attack stress has been laid on dampness. I do not propose to discuss at length this controversial subject, particularly as my own observations have led me to wonder whether after all the state of the house is of paramount importance. Some years ago we took out of the Bristol mortality records every case that could reasonably be regarded as rheumatic over a period of nearly forty years; and yet in all that mass of cases there were only eleven examples of houses in which more than one death had occurred as a result of rheumatism. In our more recent investigations of living cases we found seventeen instances of more than one in a house in a total of 754 children. This does not look as if the house were of paramount importance.

However, let us concede its occasional importance. Take, for example, the case of a family of three children, previously healthy, who shortly after moving house all developed subacute rheumatism with cardiac involvement. The obvious inference is that these children walked into an infected atmosphere, and that this accounted for their troubles. The view that a familial incidence of rheumatism can be thus explained has been stated very persuasively on a basis of evidence ingeniously derived and carefully collected by J. R. Paul, who studied a certain number of families in which two or more individuals had suffered from rheumatic fever. Amongst children between 5 and 12 years of age and previously healthy 25–35 per cent. contracted rheumatism when another member of the family was suffering from an active phase of the disease.

But three considerations bid us pause before plumping for the epidemic hypothesis. First, there has been, so far as I know, no other case of rheumatism in that house. Second, in the two schools mentioned, where saturation with the infective agent was general, rheumatic lesions developed in a small proportion only. Finally, the familial factor may be independent of environment, as in the following family. The eldest member of the family, now aged 19, developed rheumatic heart disease while living in London at the age of 6. The family later moved to Bristol, and there two younger girls were found to be suffering from the disease at the ages of 8 and 4 respectively. All the evidence points to the fact that the two younger children were infected at different times and not together.

But it is perhaps too precipitate to speak yet of a familial factor. Let us for the moment be content to admit that the development of rheumatism in a few only of those who were exposed argues the existence of an inherent factor. Before coming to closer grips with this, we must look briefly at the trend of bacteriological thought in relation to rheumatism.

Prior to the year 1900, when you, sir, with Dr. Paine first described your findings of a diplococcus in the blood and joints of rheumatic fever patients, many different workers had found almost as many different types of organisms in rheumatic lesions. The only finding to be repeated and which claimed any serious attention was that of Achalme, who grew a large gram positive bacillus from rheumatic fever patients both during life and after death. Since that time the main bacteriological interest has centred on the diplococcus or streptococcus described by Dr. Paine and yourself. Many workers have repeated your experiments with varying success, the one constant feature was that practically in every case in which an organism was found that organism was a streptococcus. In 1906 Andrewes and Horder described their sugar reactions, and showed that the diplococcus rheumaticus very closely resembled the streptococci commonly inhabiting the alimentary tract. We were then faced with the problem of how an organism of such low virulence, whose habitat was the normal alimentary canal, could produce such a virulent infection as rheumatic pancarditis. Such was the state of affairs until about 1926, the streptococcal theory gradually gaining ground in the absence of any serious opponent. About this time Swift and his colleagues in New York showed that the non-hæmolytic streptococci produced a very peculiar hypersensitive condition in rabbits. In this state minute injections of streptococci, much too small to produce any recognizable lesion in a normal animal, are followed by a marked inflammatory reaction. Further, it was found that animals sensitized to one strain would react abnormally to a different as well as to the sensitizing strain of organisms. Kaiser showed that in about 75 per cent. of children with a history of rheumatism the skin was hypersensitive to a filtrate of the an-hæmolytic streptococcus, whereas in children with no history of rheumatism not more than 35 per cent. were so sensitized. We were thus introduced to the view that rheumatism may be the response to a streptococcal infection in a patient previously sensitized to that organism. Coburn's work, which has been confirmed by Schlesinger, Sheldon and Collis, and the school epidemic I have described to you, shows that in certain cases a naso-pharyngitis caused by a hæmolytic streptococcus is followed by rheumatic manifestations after an interval of about ten to twenty-one days. Thus we see the streptococcus more and more implicated as the causal factor. Which type of streptococcus is responsible and in what manner it stimulates such a severe reaction on the part of the tissues is as yet not clear. This allergic view, however, throws some light on the predisposition of certain children and families to infection that we have already noted. Further work will show whether we are to blame the hæmolytic or non-hæmolytic strain or possibly a combination of the two. This bacteriology has an important bearing on treatment. It is difficult to see how we are to abolish such ubiquitous an organism as the streptococcus. Neither do we know of any drug exerting such an action on this group of bacteria as that of quinine on the malaria parasite. Salicylates, it is true, do influence favourably such exudative manifestations of rheumatism as the arthritis. On the proliferative lesions, such as the subcutaneous node, however, and the all-important carditis, they would appear to have little if any effect.

We turn to the other participant in the quarrel, the predisposed or affected child. How are we to assist him in his fight against the infection ? This may be considered under two headings. We may attempt to close the portal of entry against the infection, or we may assist in the resistance put up against infection when it has gained access to the body. It has long been recognized that the onset of rheumatism is frequently preceded by an acute tonsillitis. From this it has been argued that, in some cases at any rate, the tonsils are the site through which the infection enters the circulation. However, rheumatism not uncommonly makes its first appearance in children whose tonsils have already been removed. In addition tonsillectomy does not prevent a relapse of carditis in a child already affected. Thus wholesale tonsillectomy is not going to be any help to us in our efforts to prevent the disease. Dr. Reginald Miller, however, has shown that in tonsillectomized children the infection tends to be less intense, and that chorea without carditis is more common in such children. The tonsils are only one of many aggregations of lymphoid tissue along the alimentary canal, and it is probable that all are equally effective as points of entry for the offending organism. It is interesting in this connection to recall the abdominal symptoms which sometimes occur just before or at the onset of an attack of rheumatism. Older observers often referred to these, and they have been given prominence lately by the reports of Giraldi and others, and by your own observations, sir, on rheumatic appendicitis. When we turn to the patient's resistance, however, we are on more hopeful ground. Perhaps we may digress a little first to consider how the study of rheumatism as a cardiac disorder has altered our clinical picture of the disease. The acute arthritis which was the aspect of the disease first recognized presents a sudden onset with an equally sudden subsidence. Not so, however, the carditis. We have learnt to look on this as a long-drawn-out, smouldering infection, ever ready to burst into flames at the least provocation. One of the great difficulties in treating these patients is to know when the infection may be considered at an end. Its course is so insidious that the physical signs, temperature, and pulse-rates help us little. Although a definite leucocytosis accompanies the infection, it is often so low that its low limits coincide with upper limits of normal. For this reason it is unwise to place much reliance on this factor. Gain in weight we have found to be of the greatest value, as also has McCullough. More recently we have been studying the sedimentation rate of the red-blood corpuscles, and in this phenomenon it would appear that we have a most valuable guide in our treatment of rheumatic carditis. The protracted nature of the infection presents many analogies to tuberculosis. In tuberculosis we rest the affected part and build up the patient's resistance with good food, fresh air and sunshine. So, too, with rheumatic carditis; the heart cannot be rested completely, but we can see to it that as few demands as possible are made on it by keeping the patient at rest in bed for as long as active infection persists. Thus we have come to treat rheumatic children in hospital schools. Here they rest their damaged hearts, away from the detrimental environment of their homes, and in addition their minds are kept occupied and their fingers busy. Our experience of our own hospital at Winford is too recent to give you any statistics as to cures and improvements, but of its value we are certain. You may be surprised that I said cures, but rheumatic carditis does recover, at least to the extent of the disappearance of all abnormal physical signs. A fact which you, yourself, sir, have confirmed. It is this fact which encourages us to go on in the belief that one day cardiac rheumatism will be prevented.

